# Complete mitochondrial genome sequence for the *Thrips hawaiiensis* (Thysanoptera: Thripidae)

**DOI:** 10.1080/23802359.2021.1942268

**Published:** 2021-06-22

**Authors:** Yunyu Wang, Chunying Wu, Yanlan Xie, Hui Liu, Hongrui Zhang

**Affiliations:** a Plant Protection College, Yunnan Agricultural University, Kunming, China; b State Key Laboratory of Genetic Resources and Evolution, Kunming Institute of Zoology, China Academy of Sciences, Kunming, China; c College of Biotechnology and Engineering, West Yunnan University, Lincang, China

**Keywords:** *Thrips hawaiiensis*, mitochondrial genome, next-generation sequencing, Thysanoptera

## Abstract

*Thrips hawaiiensis* (Morgan) (Thysanoptera: Thripidae) is a common Thysanoptera insect widely distributed in Asia and the Pacific, it damages various plants. In this study the complete mitochondrial genome of *T. hawaiiensis* was sequenced and characterized by using next-generation sequencing technique. The total length of the complete genome is 15,357 bp and A + T content of 77.8% (GeneBank accession No. MW582621). The *T. hawaiiensis* mitochondrial genome consists of 13 protein-coding genes (PCGs), 2 ribosomal RNA genes, 22 transfer RNA genes (tRNAs) and 2 non-coding control regions (Dloop region). According to previous studies, only a few complete mitochondrial genomes from Order Thysanoptera have been reported. Thus, *T. hawaiiensis* complete mitochondrial genome sequence reported will provide molecular information for mitochondrial genome research on Thysanoptera.

*Thrips hawaiiensis*, (Thysanoptera, Thripidae) is a tiny insect, widespread across Asia and the Pacific, and has also been recorded from several locations in southern states of the USA, including Jamaica (Nakahara 1994). This species is highly polyphagous, mainly feeds on the flowers of many plants, and damages various vegetables such as melons, cowpeas, string beans, peppers, eggplants. This thrips species with many variations on morphology, body brown or bicolored (with abdomen brown and head and thorax orange-yellow) (Mound and Masumoto 2005).

In this study, the complete mitochondrial genome of *T. hawaiiensis* was sequenced successfully. The thrips samples were collected from *Ternstroemia yunnanensis* plant at Menghai, Yunnan, China (latitude 21.96 N and longitude100.64E) in 2020, subsequently identified to species by morphology. Voucher specimens (#WYY9) were deposited at Plant Protection College, Yunnan Agricultural University. Sample DNA was extracted from 30 *T. hawaiiensis* insects that come from the same place and same plant. DNA extracting kit was DNesy Blood & Tissue Kit (Qiagen, Valencia, CA). Library construction and sequencing were done with Illumina NovaSeq 6000 System in Southern China DNA Barcoding Center, State Key Laboratory of Genetic Resources and Evolution, Kunming Institute of Zoology, China Academy of Sciences. The original sequence data purified by AdapterRemoval first, after that we used Sickle to align the reference sequence. Assembling was carried out with SPAdes. Reads were assembled using Linux-OS SPAdes genome assembler v3.12.0 (Bankevich et al. 2012) with k-mer 21, 33, 55. The tRNAs sequences were confirmed using the online Search Service tRNAscan-SE (Schattner et al. 2005).

The complete mitochondrial genome of *T. hawaiiensis*is a typical closed-circular DNA molecule with a total length of 15,357 bp. The total nucleotide composition was estimated by MEGA X (Kumar et al. 2018), the overall base composition was A: 43.4%, C: 12.3%, G: 9.9%, and T: 34.4%. This genome is AT-rich in accordance with other thrips and almost all insect mitochondrial genomes (Tyagi et al. 2020). The majority strand was 77.8% A + T content and 22.2% G + C with a weakly positive AT skew (0.12) and negative GC skew (−0.11). The assembled genome was annotated by using MITOS web-server (http://mitos.bioinf.uni-leipzig.de/index.py) to estimate the position of PCGs, tRNAs, rRNAs, and their secondary structure. The genome organization consists of 37 genes, including 13 protein-coding genes (PCG), 2 ribosomal RNA (rRNAs), and 22 transfer RNA genes (tRNA), and two control regions (CRs). Most of the genes of *T. hawaiiensis* were encoded on the majority strand except three PCGs (*nad5, nad4, nad4l*) and three tRNAs (*trnY*, *trnH*, and *trnP*).

In addition, all the 13 protein-coding genes used ATN start codons (seven with ATA, five with ATT, and one with ATG) as observed in majority of the insect mitochondrial genomes (Crozier and Crozier 1993; Korkmaz et al. 2015). The stop codon TAA was used by 11 PCGs, and TAG for *cox1* and *nad4l*. The length of all 22 tRNA genes is between 59 and 69 bp. Most tRNAs have the typical cloverleaf secondary structure, however, the DHU stem and loop were absent in *trnV* and *trnS1*, while the TΨC loop was absent in *trnR* and *trnS1*. There are two control regions (CR1 and CR2) detected in *T. hawaiiensis* mitochondrial genome. We observed that the two putative control regions show more than 99% sequence similarity indicating a possible duplication. The occurrence of multiple CRs in thrips mitochondrial genomes seems to be a derived trait (Chakraborty et al. 2018) requiring further investigation.

Thrips mitochondrial genomes are marked by high rates of gene rearrangement, duplications of the control region, and tRNA mutations (Liu et al. 2017). Among order Thysanoptera, gene arrangement occurred frequently, and the mitochondrial gene arrangement in *T. hawaiiensis* differs from that of the other thrips. However the *T. hawaiiensis* protein-coding genes order same with *Thrips palmi* (Chakraborty et al. 2018) and *Thrips imagins* (Shao and Barker 2003), which have shown that in insects mitochondrial tRNA genes are much more mobile than protein-coding genes and rRNA genes (Song et al. 2016).

The total length of all 13 protein-coding genes is 11,007 bp, accounting for 71.67% of the whole genome sequence. To validate the reliability of the genome, a comparison was made with other mitochondrial genomes from Thysanoptera species. We download 13 Thysanoptera species, 14 mitochondrial genomes from GenBank first, then analyzed their amino acid sequences of 13 PCGs with neighbor-joining tree (NJ) and maximum likelihood (ML) in MEGA X. We obtained the phylogenetic position method to understand the phylogenetic relationship of *T. hawaiiensis* with other Thysanoptera insect (Figure 1). In the tree, *T. hawaiiensis* and other two *Thrips* genus species clustered into a branch, forming a sister clade with the *Franliniella* genus (Figure 1). Our study will provide a useful database for analyzing the phylogenetic relationship of *T. hawaiiensis* and other Thrips, also provide molecular information for Mitochondrial genome research on Thysanoptera.

**Figure 1.**
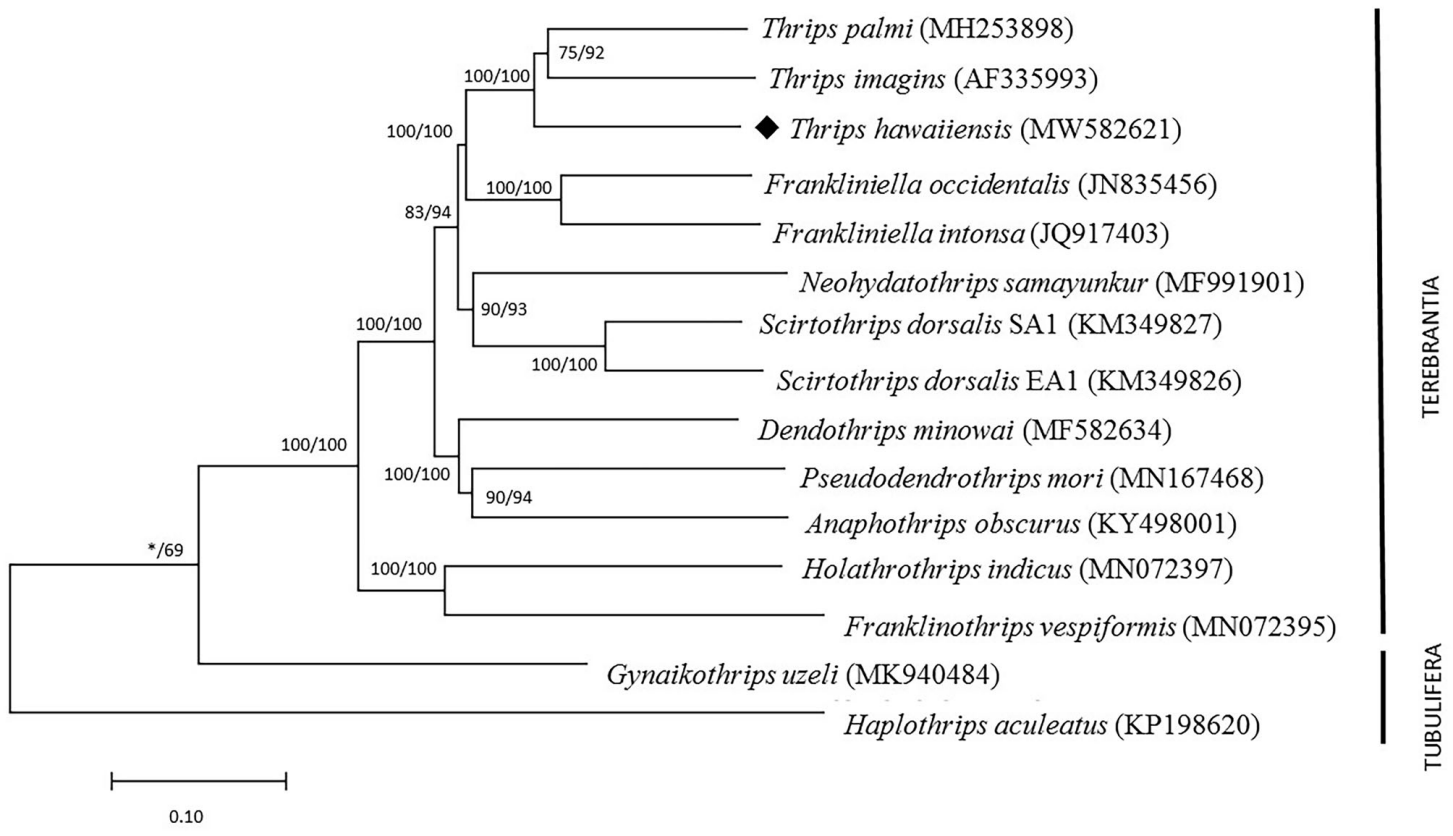
Phylogenetic relationships among Terebrantia in Thysanoptera based on mitochondrial genomes of 13 genomes with their species name and the GenBank accession numbers. Two species of Tubulifera (*Gynaikothrips uzeli* and *Haplothrips aculeatus*) were set as outgroup taxon. Number above each node indicates the NJ and ML bootstraps support values, respectively.

## Data Availability

The data that support the findings of this study are openly available in GeneBank at https://www.ncbi.nlm.nih.gov/nuccore/MW582621, reference number is No. MW582621. The associated Biproject, SRA, and BioSample nubers are PRJNA729597, SRS8959755 and SAMN19135842 respectively.
